# Fluorine-Free Super-Liquid-Repellent Surfaces: Pushing the Limits of PDMS

**DOI:** 10.1021/acs.nanolett.2c03779

**Published:** 2023-04-11

**Authors:** Katharina I. Hegner, Chirag Hinduja, Hans-Jürgen Butt, Doris Vollmer

**Affiliations:** Department of Physics at Interfaces, Max Planck Institute for Polymer Research, Ackermannweg 10, 55128 Mainz, Germany

**Keywords:** Friction, Nanoparticles, Superhydrophobic, Surface chemistry, Wetting

## Abstract

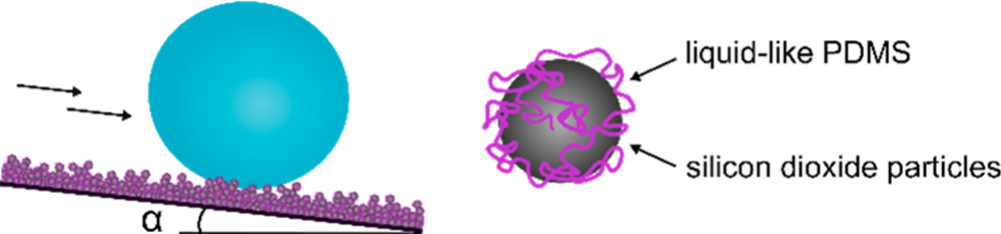

Methods for fabricating super-liquid-repellent surfaces have typically relied on perfluoroalkyl substances. However, growing concerns about the environmental and health effects of perfluorinated compounds have caused increased interest in fluorine-free alternatives. Polydimethylsiloxane (PDMS) is most promising. In contrast to fluorinated surfaces, PDMS-coated surfaces showed only superhydrophobicity. This raises the question whether the poor liquid repellency is caused by PDMS interacting with the probe liquid or whether it results from inappropriate surface morphology. Here, we demonstrate that a well-designed two-tier structure consisting of silicon dioxide nanoparticles combined with surface-tethered PDMS chains allows super-liquid-repellency toward a range of low surface tension liquids. Drops of water–ethanol solutions with surface tensions as low as 31.0 mN m^–1^ easily roll and bounce off optimized surface structures. Friction force measurements demonstrate excellent surface homogeneity and easy mobility of drops. Our work shows that fluorine-free super-liquid-repellent surfaces can be achieved using scalable fabrication methods and environmentally friendly surface functionalization.

Inspired by structures found in various plants^1^ and insects,^2,3^ artificial super-liquid-repellent surfaces have been developed for applications including self-cleaning,^4^ heat transfer,^5^ anti-icing,^6^ or antibiofouling.^7,8^ On super-liquid-repellent surfaces, drops exhibit apparent contact angles exceeding 150° and roll off when the surface is tilted by less than 10°.^9,10^ In order to achieve this, appropriate surface structures combined with low surface energy are required.^11,12^ Perfluoroalkyl substances are conventionally used to achieve super-liquid-repellency toward low surface tension liquids,^13,14^ yet there are growing concerns toward this class of chemicals. They are persistent and may bioaccumulate in humans, animals, and plants,^15−17^ thereby posing a danger to human health such as thyroid disease, liver damage, and effects on reproduction and fertility.^17^ Therefore, fluorine-free alternatives are urgently needed.

Various strategies have been tested to design fluorine-free surfaces, where droplets of water and low surface tension liquids easily roll off.^18^ Silicones are considered promising although super-liquid-repellency against low surface tension liquids has not yet been achieved. There are multiple benefits to silicones; they are nontoxic, biocompatible, colorless, and cheap. Most importantly, they have a low surface energy of approximately 20 mN m^–1^.^19^ The most common silicone is trimethylsiloxyl-terminated linear poly(dimethylsiloxane) (PDMS). On flat surfaces functionalized with PDMS, aqueous and organic liquids roll off at low tilt angles.^20−24^ However, the velocity of drop mobility is typically a few orders of magnitude below those on superhydrophobic surfaces. Superhydrophobic PDMS surfaces have been prepared successfully by coating preformed structures^18,25^ or through one-step processes such as the formation of fibrous silicone nanofilaments.^26^ Since water has a high surface tension of 72 mN m^–1^, superhydrophobicity is relatively easy to achieve. The lower the surface tension of the liquid, the more difficult it becomes to engineer suitable surface morphologies supporting drops in the Cassie state.^11,12^ To the best of our knowledge, so far repellency toward ethylene glycol (γ_EG_ = 47.7 mN m^–1^) has only been achieved by the partial decomposition of PDMS at elevated temperatures.^27^ The results raise the question of whether this is a fundamental limit caused by the interactions between PDMS and the probe liquid, or whether super-liquid-repellency toward lower surface tension liquids can be achieved using suitable surface morphologies.

In this work, we demonstrate that the poor wetting properties of previously reported surfaces result from the surface morphology. We present surfaces that are able to repel liquids with surface tensions as low as 31.0 mN m^–1^, i.e., liquids having a surface tension more than 15 mN m^–1^ lower than previously reported data. Contact angles above 150°, low roll-off angles below 10°, and rebound of impacting drops ensure high drop mobility. The surfaces consist of silicon dioxide nanoparticles that are functionalized with surface-tethered PDMS chains. We demonstrate that a well-designed roughness on the nano- and micrometer scale allows fluorine-free super-liquid-repellency.

To investigate the significance of surface morphology and overhang morphologies on the nano- and microscale on fluorine-free super-liquid-repellency, we prepared a range of silicon dioxide particle-based surfaces. Three fabrication methods, namely liquid flame spray, spray coating, and the deposition of soot from a paraffin candle were employed (Figure 1a–d and Supporting Information, Methods for experimental details, and Figure S3a–d for top-view scanning electron microscopy images).

**Figure 1:**
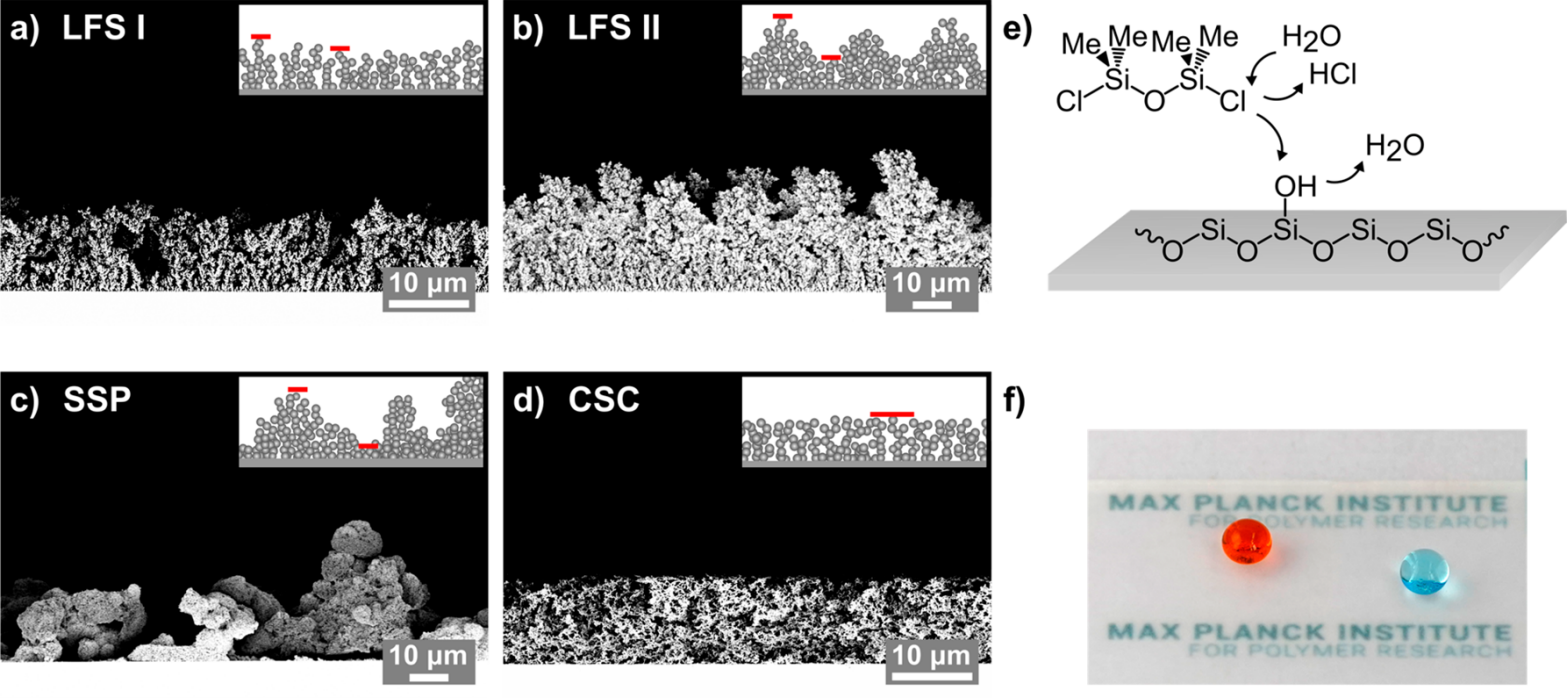
Scanning electron microscopy images of various particle-based surfaces: (a) liquid flame spray I (LFS I), (b) liquid flame spray II (LFS II), (c) sprayed silica particles (SSP), and (d) a candle soot coating (CSC). The insets show schematic illustrations of the particle structures with the red lines indicating the structural differences in height. (e) Functionalization with dichlorotetramethyldisiloxane results in the formation of surface-tethered linear PDMS chains which render the surfaces super-liquid-repellent. (f) Optical images of dyed 15 μL droplets of water (blue) and ethylene glycol (orange) on a PDMS-functionalized SSP surface.

In liquid flame spray (LFS), a liquid feed containing the precursor (tetraethyl orthosilicate in isopropanol) was injected into a CH_4_/O_2_ flame where the feed evaporates and the precursor combusts. Silica particles nucleate and grow. The time and process distance at which nanoparticles are collected determine their diameter, degree of aggregation, and the coating’s microscale roughness.^28^ A thin silica shell of approximately 10 nm was added by chemical vapor deposition to enhance the mechanical stability of the coatings. For the sample LFS I, coated for 3 min at a 15 cm distance from the burner, nanoparticles with diameters of 83 ± 19 nm are aggregated into structures with heights between approximately 8 and 10 μm (Figure 1a, Inset red lines). The sample LFS II was coated for 2 min at a distance of 10 cm and displays porous protrusions that vary in height by up to 5 μm. Neighboring surface protrusions are spaced out by approximately 10 to 20 μm. The lower process distance causes reduced growth times resulting in smaller nanoparticle diameters of 47 ± 8 nm (Figure 1b).

The second type of sample was prepared by spray coating fumed silica particles with a diameter of 7 nm. The particles were dispersed in acetone (5 mg mL^–1^) and sprayed onto the substrates at a flow rate of 0.2 mL s^–1^ at 2 bar and 10 cm distance. During spray coating and drying, the particles form aggregates with a diameter of 32 ± 5 nm. These aggregates assemble into porous features with heights varying by up to 20 μm and distances of up to several tens of micrometers (Figure 1c). The sprayed silica particle (SSP) surface exhibits the highest degree of micrometer scale roughness.

Candle soot coatings (CSC) were formed from the combustion of paraffin wax in a flame.^29^ The soot deposit is coated with a silica shell to introduce the surface hydroxyl groups necessary for surface functionalization. The resulting nanoparticles have a diameter of 85 ± 18 nm. The surfaces are homogeneous on the microscale; the height varies by less than 1 μm (Figure 1d).

If not stated otherwise, the surfaces are functionalized with linear PDMS chains via solvent-free chemical vapor deposition of dichlorotetramethyldisiloxane at ambient environment (Figure 1f, Supporting Information, Methods for details on the sample preparation).^22,30^ The chlorosilane dimer is hydrolyzed by water from the environment and reacts with hydroxyl groups via condensation reactions (Figure 1e). This reaction can occur between several species including the surface hydroxyl groups, the hydrolyzed dimers, surface-grafted PDMS chains, and loose oligomers. On flat surfaces, a two-dimensional PDMS coating with a thickness of a few nanometers is formed.^20,21,23^

To quantify the super-liquid-repellency we used water–ethanol solutions. Increasing the fraction of ethanol allowed us to gradually reduce the surface tension (Supporting Information, Table T2 for surface tensions and contact angles). For all samples, the contact angles of 6 μL drops of milli-Q water exceed 165° (Figure 2a). Notably, both liquid flame spray surfaces, LFS I-PDMS (green) and LFS II-PDMS (orange) even exhibit contact angles of more than 150° for water–ethanol solutions with a surface tension of 31.0 and 32.8 mN m^–1^, respectively. Impacting drops bounce off (Supplementary Video M4). We observed roll-off angles of less than 10° for all test liquids (Figure 2b, Supplementary Video M1). The high contact and low roll-off angles are in line with a stable Cassie state, where the liquids rest on a composite solid–air interface.^31,32^ On the sprayed silica particle surface (blue), contact angles abruptly dropped to less than 100° for surface tensions lower than 34.8 mN m^–1^. While the candle soot coating (red) could repel liquids with surface tensions down to 32.8 mN m^–1^, the contact angles were continuously lower than the values observed using the LFS surfaces.

**Figure 2:**
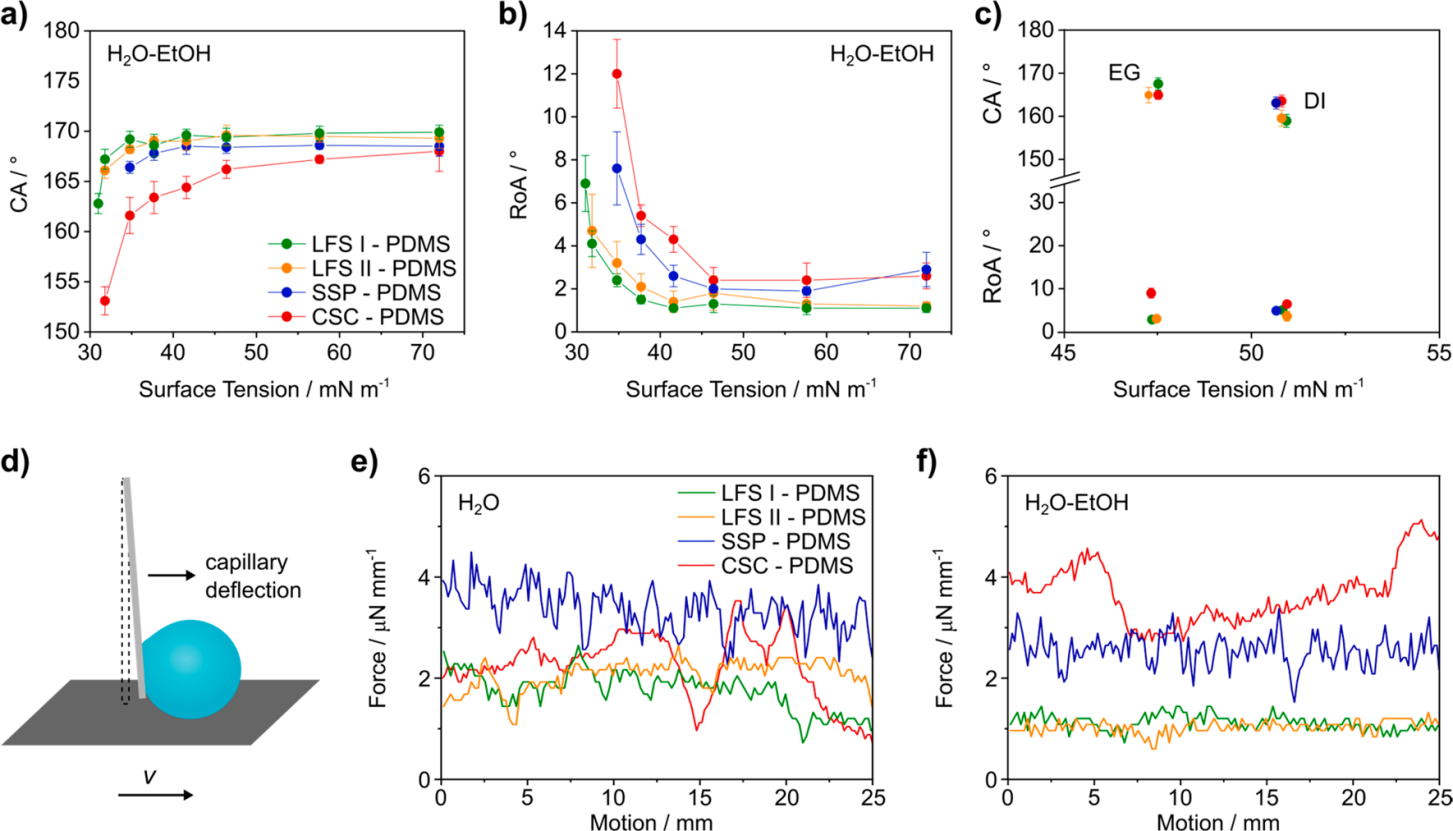
Wetting properties of structured PDMS-functionalized surfaces prepared by liquid flame spray (LFS), sprayed silica particles (SSP), and candle soot coatings (CSC). (a) Contact angle and (b) roll-off angle measurements using 6 μL droplets of water and water–ethanol solutions. The amount of ethanol was increased from 0 to 35 wt % in steps of 5 wt %. Surface tensions were measured using the pendant drop method. (c) Contact (▲) and roll-off (●) angle measurements using 6 μL droplets of ethylene glycol (EG) and diiodomethane (DI). Drop friction measurements: (d) A thin glass capillary is brought into contact with the drop (*V* = 15 μL). The surface is then moved laterally at a constant velocity (*v*) of 0.5 mm s^–1^. The deflection of the glass capillary is measured using an optical microscope camera. Drop friction measurements using (e) water and (f) a 20 wt % water–ethanol solution.

For diiodomethane (γ_DI_ = 50.9 mN m^–1^) roll-off angles between 3° and 6° and contact angles well above 150° were observed for all samples (Figure 2c). Drops of ethylene glycol (γ_EG_ = 47.7 mN m^–1^) rolled off both LFS surfaces (green, orange) at a 2° to 3° tilt.

Overall, superior super-liquid-repellency was observed for the LFS I-PDMS surface, which is able to repel water–ethanol solutions with a surface tension of only 31.0 mN m^–1^. This suggests that a porous two-tier structure with protrusions varying by a few micrometers in height is most appropriate. Liquids with even lower surface tensions such as hexadecane (γ_HD_ = 27.6 mN m^–1^) wet the surface. The Cassie–Wenzel transition may be due to the low surface tension, an enhanced affinity to the PDMS layer, or a combination thereof.

To resolve pinning of the three-phase contact line and detect spatial heterogeneities even on the micrometer scale we directly measured the friction force a drop experiences during motion.^33−37^ The friction force is determined from the deflection of a flexible glass capillary while the substrate velocity was kept constant at *v* = 0.5 mm s^–1^: *F*_F_ = *k*_s_·Δ*x* (Figure 2d). Here, Δ*x* is the deflection and *k*_s_ the spring constant of the capillary (Supporting Information, Methods for details on the friction measurements and the normalization of the data). Drop friction depends on the solid–liquid interfacial area and therefore, on the drop volume. Hence, we normalized the force to the drop radius (*r* = 1.5 mm from *V* = 15 mm^3^).

Using water, the friction forces measured on the LFS I-PDMS surface were as low as 1.8 ± 0.4 μN mm^–1^, varying by 20% across the investigated distance (Figure 2e, Supplementary Video M2). For LFS II-PDMS, the average force increased by 17% to 2.1 ± 0.3 μN mm^–1^. This is in line with a slight deterioration of the wetting properties compared to the LFS I-PDMS sample (Figure 2a). On the SSP-PDMS surface, a stick–slip motion was observed resulting in variations of the friction force of up to ±30%. The pronounced roughness results in localized pinning sites. On the candle soot-based surface the force measurements reveal noticeable surface inhomogeneities on the millimeter scale, caused by the less-controlled sooting process. Depending on the surface, the friction forces agree within 60 to 115% with friction forces calculated from the integration of the horizontal component of the liquid–air surface tension γ along the three-phase contact line^36^ (Supporting Information, Table 1 for calculated values for drop friction).

Using a water–ethanol solution (γ = 37.7 mN m^–1^), the normalized friction force decreases on both LFS by approximately a factor of 2, i.e., proportional to the decrease of the interfacial tension (Figure 2f, Supplementary Video M3). The homogeneity of the surfaces is reflected in the smooth shape of the force curves. The candle soot-based surface exhibits the highest droplet friction with 3.7 ± 0.59 μN mm^–1^. The larger friction of the water–ethanol mixture on the candle soot coating hints that the lower contact angle (Figure 2a) and likely larger contact width cause the increase of the normalized friction force (Supporting Information, Methods for details on the friction measurements).

Both, the apparent contact angle and the friction force measurements show that the LFS I surface, followed closely by the LFS II surface, exhibits the most suitable surface morphology for supporting drops in the Cassie state without having to rely on the use of perfluoroalkyl substances. A comparatively homogeneous microscale morphology increases the solid–liquid interface, thereby reducing the super-liquid repellency (CSC, Figure 1d). On the other hand, a pronounced roughness on the micrometer scale induces pinning sites which increase droplet friction and roll-off angles (SSP, Figure 1c).

So far, structures functionalized with perfluoroalkyl substances are the benchmark in the preparation of super-liquid-repellent surfaces. To investigate the influence of surface morphology on the performance of fluorinated surfaces, we functionalized the four particle-based structures with perfluorooctyltrichlorosilane (PFOTS) via chemical vapor deposition (Figure 3a, b and Supporting Information Table T3). In combination with PFOTS, all four surface morphologies are able to support hexadecane (γ_HD_ = 27.6 mN m^–1^) in the Cassie state. Nevertheless, we observe superior super-liquid-repellency using the LFS surfaces with roll-off angles of only 3° to 4°. The highest roll-off angles are observed for the candle soot-based surface. These results support the assumption that the differences in super-liquid-repellency of PDMS-functionalized surfaces are indeed due to the underlying particle structures.

**Figure 3:**
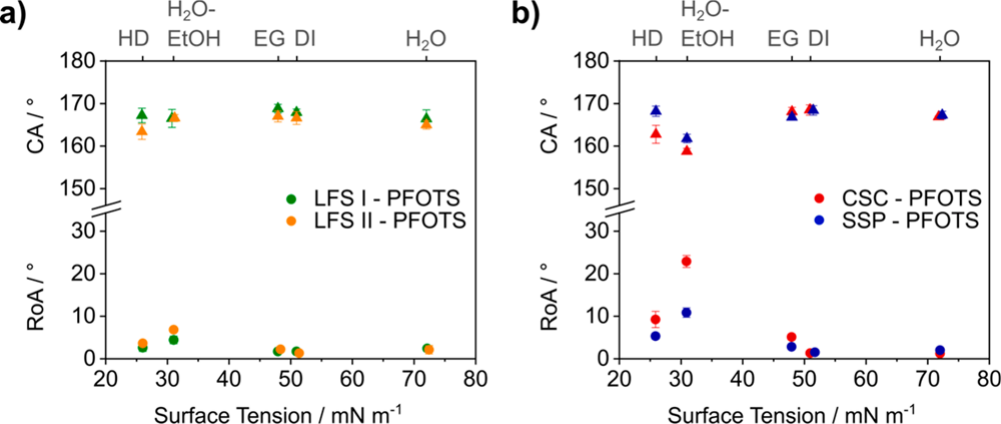
Contact angle (▲) and roll-off angle (●) measurements on structured perfluorooctyltrichlorosilane-functionalized surfaces prepared by a) liquid flame spray (LFS) and b) sprayed silica particles (SSP) and candle soot coating (CSC). The wetting properties were measured using water (γ_H_2_O_ = 72.0 mN m^–1^), diiodomethane (γ_DI_ = 50.9 mN m^–1^), ethylene glycol (γ_EG_ = 47.7 mN m^–1^), a 35 wt % aqueous ethanol solution (γ_H_2_O-EtOH_ = 31.0 mN m^–1^), and hexadecane (γ_HD_ = 27.6 mN m^–1^).

Fluorine-free super-liquid-repellency towards a range of low surface tension liquids can be achieved using PDMS. The poor wetting properties of previously prepared surfaces result from poorly designed surface morphologies rather than a conceptual limitation to the use of PDMS. However, achieving super-liquid-repellency is much more challenging than using fluorination. Likely increased affinity of organic solvents with the probe liquid causes slight swelling of the PDMS chains, resulting in an increased effective interfacial tension between PDMS and the probe liquid. Therefore, a suitable surface morphology is even more essential. Surfaces prepared via liquid flame spray show the suitable combination of dual-scale morphology with overhang structures.

Porous structures on the nano- and micrometer scale exhibit inherently low mechanical robustness. Although most applications require high mechanical stability, this does not hold in general. Comparatively low mechanical robustness is demanded, for example, in the application as membranes in water–oil separation^38^ and CO_2_ capturing,^39,40^ in (photo)electrocatalysis,^41^ and drop transport.^42^

With concerns about the bioaccumulability and toxicity of perfluoroalkyl substances on the rise, these results point a way toward fluorine-free alternatives based on a suitable surface morphology and a PDMS functionalization.

## Supporting Information Available

Methods and supporting figures and tables (PDF)
Video M1: Roll-off angle measurements with 6 μL drops of milli-Q water on LFS, SSP, and CSC surfaces (AVI)
Video M2: Friction force measurements with 15 μL drops of milli-Q water on LFS, SSP, and CSC surfaces (AVI)
Video M3: Friction force measurements with 15 μL drops of aqueous ethanol (20 wt %) on LFS, SSP, and CSC surfaces (AVI)
Video M4: Drop impact using milli-Q water (4 μL) and aqueous ethanol (35 wt %, 2.3 μL) on a LFS I surface. 0.006× speed (AVI)

## Supplementary Material

nl2c03779_si_001.pdf

nl2c03779_si_002.avi

nl2c03779_si_003.avi

nl2c03779_si_004.avi

nl2c03779_si_005.avi

## References

[ref1] Barthlott W.; Neinhuis C. Purity of the Sacred Lotus, or Escape from Contamination in Biological Surfaces. Planta 1997, 202 (1), 1–8. 10.1007/s004250050096.

[ref2] Wagner T.; Neinhuis C.; Barthlott W. Wettability and Contaminability of Insect Wings as a Function of Their Surface Sculptures. Acta Zool. 1996, 77 (3), 213–225. 10.1111/j.1463-6395.1996.tb01265.x.

[ref3] Gao X.; Jiang L. Water-Repellent Legs of Water Striders. Nature 2004, 432, 36 10.1038/432036a. 15525973

[ref4] Fürstner R.; Barthlott W.; Neinhuis C.; Walzel P. Wetting and Self-Cleaning Properties of Artificial Superhydrophobic Surfaces. Langmuir 2005, 21 (3), 956–961. 10.1021/la0401011. 15667174

[ref5] Miljkovic N.; Wang E. N. Condensation Heat Transfer on Superhydrophobic Surfaces. MRS Bull. 2013, 38, 397 10.1557/mrs.2013.103.

[ref6] Kreder M. J.; Alvarenga J.; Kim P.; Aizenberg J. Design of Anti-Icing Surfaces: Smooth, Textured or Slippery?. Nat. Rev. Mater. 2016, 1 (1), 15003 10.1038/natrevmats.2015.3.

[ref7] Encinas N.; Yang C. Y.; Geyer F.; Kaltbeitzel A.; Baumli P.; Reinholz J.; Mailänder V.; Butt H. J.; Vollmer D. Submicrometer-Sized Roughness Suppresses Bacteria Adhesion. ACS Appl. Mater. Interfaces 2020, 12 (19), 21192–21200. 10.1021/acsami.9b22621. 32142252 PMC7226781

[ref8] Geyer F.; D’Acunzi M.; Yang C. Y.; Müller M.; Baumli P.; Kaltbeitzel A.; Mailänder V.; Encinas N.; Vollmer D.; Butt H. J. How to Coat the Inside of Narrow and Long Tubes with a Super-Liquid-Repellent Layer—A Promising Candidate for Antibacterial Catheters. Adv. Mater. 2019, 31 (2), 1801324 10.1002/adma.201801324. 30417451

[ref9] Quéré D. Wetting and Roughness. Annu. Rev. Mater. Res. 2008, 38, 71–99. 10.1146/annurev.matsci.38.060407.132434.

[ref10] Pan S.; Guo R.; Björnmalm M.; Richardson J. J.; Li L.; Peng C.; Bertleff-Zieschang N.; Xu W.; Jiang J.; Caruso F. Coatings Super-Repellent to Ultralow Surface Tension Liquids. Nat. Mater. 2018, 17 (11), 1040–1047. 10.1038/s41563-018-0178-2. 30323333

[ref11] Butt H. J.; Semprebon C.; Papadopoulos P.; Vollmer D.; Brinkmann M.; Ciccotti M. Design Principles for Superamphiphobic Surfaces. Soft Matter 2013, 9 (2), 418–428. 10.1039/C2SM27016A.

[ref12] Tuteja A.; Choi W.; Ma M.; Mabry J. M.; Mazzella S. A.; Rutledge G. C.; Mckinley G. H.; Cohen R. E. Designing Superoleophobic Surfaces. Science 2007, 318, 1618–1622. 10.1126/science.1148326. 18063796

[ref13] Paxson A. T.; Varanasi K. K. Self-Similarity of Contact Line Depinning from Textured Surfaces. Nat. Commun. 2013, 4, 1–8. 10.1038/ncomms2482. PMC3586717 23422660

[ref14] Peng C.; Chen Z.; Tiwari M. K. All-Organic Superhydrophobic Coatings with Mechanochemical Robustness and Liquid Impalement Resistance. Nat. Mater. 2018, 17 (4), 355–360. 10.1038/s41563-018-0044-2. 29581573

[ref15] Scheringer M.; Trier X.; Cousins I. T.; de Voogt P.; Fletcher T.; Wang Z.; Webster T. F. Helsingør Statement on Poly- and Perfluorinated Alkyl Substances (PFASs). Chemosphere 2014, 114, 337–339. 10.1016/j.chemosphere.2014.05.044. 24938172

[ref16] Conder J. M.; Hoke R. A.; de Wolf W.; Russell M. H.; Buck R. C. Are PFCAs Bioaccumulative? A Critical Review and Comparison with Regulatory Criteria and Persistent Lipophilic Compounds. Environ. Sci. Technol. 2008, 42 (4), 995–1003. 10.1021/es070895g. 18351063

[ref17] European Commission. Communication from the commission to the European parliament, the council, the European economic and social committee and the committee of the regions. Poly- and perfluoroalkyl substances (PFAS): Chemicals Strategy for Sustainability Towards a Toxic-Free Environment, 2020. https://ec.europa.eu/environment/pdf/chemicals/2020/10/SWD_PFAS.pdf. (accessed 2023-02-01).

[ref18] Wang Q.; Sun G.; Tong Q.; Yang W.; Hao W. Fluorine-Free Superhydrophobic Coatings from Polydimethylsiloxane for Sustainable Chemical Engineering: Preparation Methods and Applications. Chem. Eng. J. 2021, 426, 130829 10.1016/j.cej.2021.130829.

[ref19] Colas A. Silicones: Preparation, Properties and Performance; Elsevier, Amsterdam, 2005.

[ref20] Wang L.; McCarthy T. J. Covalently Attached Liquids: Instant Omniphobic Surfaces with Unprecedented Repellency. Angew. Chem., Int. Ed. 2016, 55 (1), 244–248. 10.1002/anie.201509385. 26568536

[ref21] Teisala H.; Baumli P.; Weber S. A. L.; Vollmer D.; Butt H. J. Grafting Silicone at Room Temperature - a Transparent, Scratch-Resistant Nonstick Molecular Coating. Langmuir 2020, 36 (16), 4416–4431. 10.1021/acs.langmuir.9b03223. 32239949 PMC7191751

[ref22] Zhao X.; Khandoker M. A. R.; Golovin K. Non-Fluorinated Omniphobic Paper with Ultralow Contact Angle Hysteresis. ACS Appl. Mater. Interfaces 2020, 12 (13), 15748–15756. 10.1021/acsami.0c01678. 32142254

[ref23] Liu J.; Sun Y.; Zhou X.; Li X.; Kappl M.; Steffen W.; Butt H. J. One-Step Synthesis of a Durable and Liquid-Repellent Poly(Dimethylsiloxane) Coating. Adv. Mater. 2021, 33 (23), 2100237 10.1002/adma.202100237. PMC11468872 33955585

[ref24] Chen W.; Fadeev A. Y.; Hsieh M. C.; Öner D.; Youngblood J.; McCarthy T. J. Ultrahydrophobic and Ultralyophobic Surfaces: Some Comments and Examples. Langmuir 1999, 15 (10), 3395–3399. 10.1021/la990074s.

[ref25] Wu S.; Du Y.; Alsaid Y.; Wu D.; Hua M.; Yan Y.; Yao B.; Ma Y.; Zhu X.; He X. Superhydrophobic Photothermal Icephobic Surfaces Based on Candle Soot. Proc. Natl. Acad. Sci. U.S.A. 2020, 117 (21), 11240–11246. 10.1073/pnas.2001972117. 32393646 PMC7260993

[ref26] Artus G. R. J.; Jung S.; Zimmermann J.; Gautschi H. P.; Marquardt K.; Seeger S. Silicone Nanofilaments and Their Application as Superhydrophobic Coatings. Adv. Mater. 2006, 18 (20), 2758–2762. 10.1002/adma.200502030.

[ref27] Long M.; Peng S.; Yang X.; Deng W.; Wen N.; Miao K.; Chen G.; Miao X.; Deng W. One-Step Fabrication of Non-Fluorinated Transparent Super-Repellent Surfaces with Tunable Wettability Functioning in Both Air and Oil. ACS Appl. Mater. Interfaces 2017, 9 (18), 15857–15867. 10.1021/acsami.7b01926. 28426195

[ref28] Hegner K. I.; Wong W. S. Y.; Vollmer D. Ultrafast Bubble Bursting by Superamphiphobic Coatings. Adv. Mater. 2021, 33 (39), 2101855 10.1002/adma.202101855. PMC11468632 34365676

[ref29] Deng X.; Mammen L.; Butt H. J.; Vollmer D. Candle Soot as a Template for a Transparent Robust Superamphiphobic Coating. Science 2012, 335 (6064), 67–70. 10.1126/science.1207115. 22144464

[ref30] Shabanian S.; Khatir B.; Nisar A.; Golovin K. Rational Design of Perfluorocarbon-Free Oleophobic Textiles. Nat. Sustain. 2020, 3 (12), 1059–1066. 10.1038/s41893-020-0591-9.

[ref31] Cassie A. B. D. Contact Angles. Discuss. Faraday Soc. 1948, 3, 11 10.1039/df9480300011.

[ref32] Cassie A. B. D.; Baxter S. Wettability of Porous Surfaces. Trans. Faraday Soc. 1944, 40, 546 10.1039/tf9444000546.

[ref33] Hokkanen M. J.; Backholm M.; Vuckovac M.; Zhou Q.; Ras R. H. A. Force-Based Wetting Characterization of Stochastic Superhydrophobic Coatings at Nanonewton Sensitivity. Adv. Mater. 2021, 33 (42), 2105130 10.1002/adma.202105130. PMC11468561 34469006

[ref34] Daniel D.; Timonen J. V. I.; Li R.; Velling S. J.; Kreder M. J.; Tetreault A.; Aizenberg J. Origins of Extreme Liquid Repellency on Structured, Flat, and Lubricated Hydrophobic Surfaces. Phys. Rev. Lett. 2018, 120 (24), 1–5. 10.1103/PhysRevLett.120.244503. 29956993

[ref35] Pilat D. W.; Papadopoulos P.; Schäffel D.; Vollmer D.; Berger R.; Butt H. J. Dynamic Measurement of the Force Required to Move a Liquid Drop on a Solid Surface. Langmuir 2012, 28 (49), 16812–16820. 10.1021/la3041067. 23181385

[ref36] Gao N.; Geyer F.; Pilat D. W.; Wooh S.; Vollmer D.; Butt H. J.; Berger R. How Drops Start Sliding over Solid Surfaces. Nat. Phys. 2018, 14 (2), 191–196. 10.1038/nphys4305.

[ref37] Furmidge C. G. L. Studies at Phase Interfaces. I. The Sliding of Liquid Drops on Solid Surfaces and a Theory for Spray Retention. J. Colloid Sci. 1962, 17 (4), 309–324. 10.1016/0095-8522(62)90011-9.

[ref38] Guo W.; Wang X.; Huang J.; Zhou Y.; Cai W.; Wang J.; Song L.; Hu Y. Construction of Durable Flame-Retardant and Robust Superhydrophobic Coatings on Cotton Fabrics for Water-Oil Separation Application. Chem. Eng. J. 2020, 398, 125661 10.1016/j.cej.2020.125661.

[ref39] Geyer F.; Schönecker C.; Butt H.-J.; Vollmer D. Enhancing CO2 Capture Using Robust Superomniphobic Membranes. Adv. Mater. 2017, 29 (5), 1603524 10.1002/adma.201603524. 27896855

[ref40] Baidya A.; Yatheendran A.; Ahuja T.; Sudhakar C.; Das S. K.; Ras R. H. A.; Pradeep T. Waterborne Fluorine-Free Superhydrophobic Surfaces Exhibiting Simultaneous CO2 and Humidity Sorption. Adv. Mater. Interfaces 2019, 6 (23), 1901013 10.1002/admi.201901013.

[ref41] Liu G.; Wong W. S. Y.; Kraft M.; Ager J. W.; Vollmer D.; Xu R. Wetting-Regulated Gas-Involving (Photo)Electrocatalysis: Biomimetics in Energy Conversion. Chem. Soc. Rev. 2021, 50 (18), 10674–10699. 10.1039/D1CS00258A. 34369513

[ref42] Mertaniemi H.; Jokinen V.; Sainiemi L.; Franssila S.; Marmur A.; Ikkala O.; Ras R. H. A. Superhydrophobic Tracks for Low-Friction, Guided Transport of Water Droplets. Adv. Mater. 2011, 23 (26), 2911–2914. 10.1002/adma.201100461. 21538591

