# Supplementary material for: Fluorine-Free Super-Liquid-Repellent Surfaces: Pushing the Limits of PDMS
Source: Nano Lett. 2023 Apr 11;23(8):3116–21. doi: 10.1021/acs.nanolett.2c03779 (PMC10141414; doi:10.1021/acs.nanolett.2c03779)
Supplement: Supplementary file 1 — nl2c03779_si_001.pdf [file nl2c03779_si_001.pdf]

Supporting Information

Fluorine-Free Super-Liquid-Repellent Surfaces: Pushing the Limits of PDMS

Katharina I. Hegner, Chirag Hinduja, Hans-Jürgen Butt, Doris Vollmer*

This file includes:

Methods, Supporting Figures S1 to S7, and Supporting Tables T1 to T3

Other Supplementary Materials for this manuscript include the following:

Supplementary Video M1 – 6 μL drops of milli-Q water rolling off PDMS-functionalized surfaces tilted at 0.5 degree per second. LFS: liquid flame spray; SSP: sprayed silica particles; CSC: candle soot coating. The video shows an area of approximately 7 mm.

Supplementary Video M2 – Friction force measurements using 15 μL drops of milli-Q water. Drops are moved across PDMS-functionalized surfaces at a constant velocity of 0.5 mm per second. LFS: liquid flame spray; SSP: sprayed silica particles; CSC: candle soot coating.

Supplementary Video M3 – Friction force measurements using 15 μL drops of a water-ethanol solution (20 wt% EtOH, $\gamma = 37.7$ mN m^{-1}). Drops are moved across PDMS-functionalized surfaces at a constant velocity of 0.5 mm per second. LFS: liquid flame spray; SSP: sprayed silica particles; CSC: candle soot coating.

Supplementary Video M4 – Drop impact using milli-Q water (4 μL) and a water-ethanol solution (2.3 μL, 35 wt% EtOH, $\gamma = 31.0$ mN m^{-1}) on a LFS I sample functionalized with PDMS and PFOTS, slowed down to 0.006x speed. The substrate was tilted by 15 degree. LFS: liquid flame spray.

Methods

Synthesis:

Silicon dioxide particle-based coatings were prepared via liquid flame spray (LFS), spray coating (SSP) and candle soot (CSC), Figure S2.

For liquid flame spray (Figure S1), a pilot flame was created by the process gases oxygen (2 L min^{-1}) and methane (1 L min^{-1}). A liquid feedstock consisting of tetraethyl orthosilicate (98 %, Sigma Aldrich) in isopropanol (Fisher Scientific) was dispersed into the flame. As a dispersion gas, oxygen was used at a flowrate of 5 L min^{-1}. The concentration of the

precursor in isopropanol was 370 mg mL^{-1}. The particles were collected on glass substrates (Thermo Scientific) at a distance of 10 cm and 15 cm with respect to the burner unit for 2 min and 3 min, respectively. A silica shell was added via chemical vapor deposition to enhance the mechanical stability of the coatings. The surfaces were placed in a desiccator together with tetraethyl orthosilicate (98 %, Sigma Aldrich, 1 mL in 2.400 cm^3) and aqueous ammonia solution (25 %, VWR

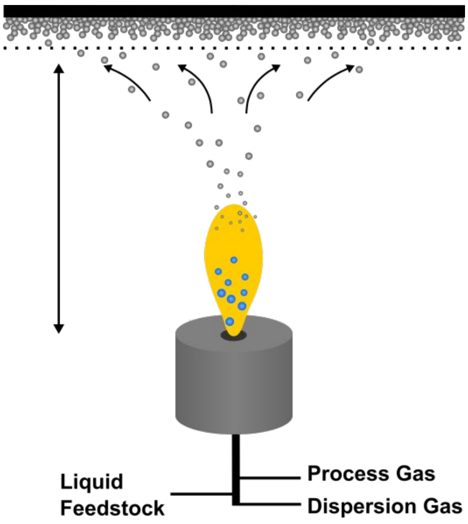

Figure S1. Schematic illustration of the LFS

Chemicals, 1 mL in 2.400 cm^3). The reaction was allowed to proceed for 16 h at atmospheric pressure. Afterwards, the coatings were sintered for 3 h at 500 ˚C in air. Higher sintering temperatures result in a decrease in surface roughness (Figure S2).

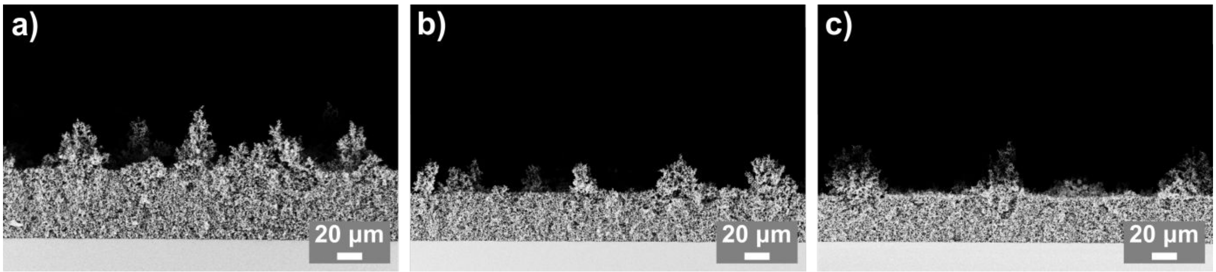

Figure S2. SEM images of LFS films deposited onto silicon wafers exposed to a) no sintering, b) sintering at 500˚C, and c) sintering at 1000˚C. Sintering at higher temperatures results in an overall reduction in surface roughness.

For the samples prepared via spray coating, fumed silicon dioxide nanoparticles (Aldrich, 7 nm, SSA = 395 m^2 g^{-1}) were dispersed in acetone at a concentration of 5 mg mL^{-1}. 5 mL of the dispersion were used to coat a microscope glass slide (Thermo Scientific) with an area of 76 x 26 mm. Samples were sprayed with a flow rate of 0.2 mL s^{-1} at 2 bar at a distance of 10 cm using a spray gun with a nozzle diameter with 0.5 mm. Afterwards, the samples were allowed to dry for 24 h. The candle soot-based particle surfaces were prepared according to a procedure described by Deng et al.[1]

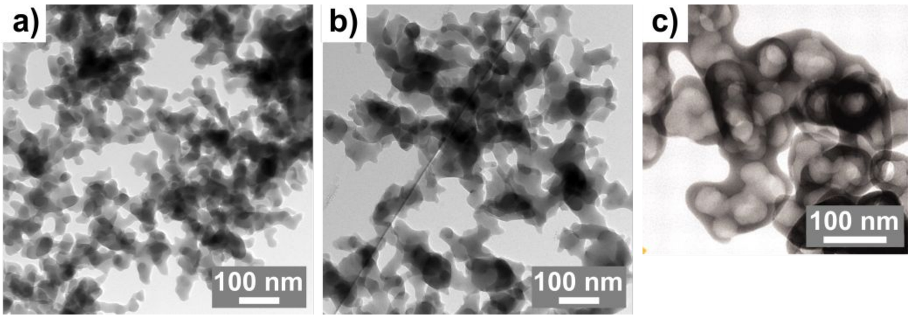

Figure S3. TEM images of silica particles prepared via liquid flame spray at a distance of a) 15 cm and b) 10 cm. c) TEM image of a candle soot templated silica particle cluster after calcination. Reprinted (Adapted or Reprinted in part) with permission from Deng et al.[1] Copyright 2011 Science published by The American Association for the Advancement of Science. In contrast to the LFS particle clusters, the candle soot templated silica particles are hollow. The particle clusters are stabilized by a thin silica shell.

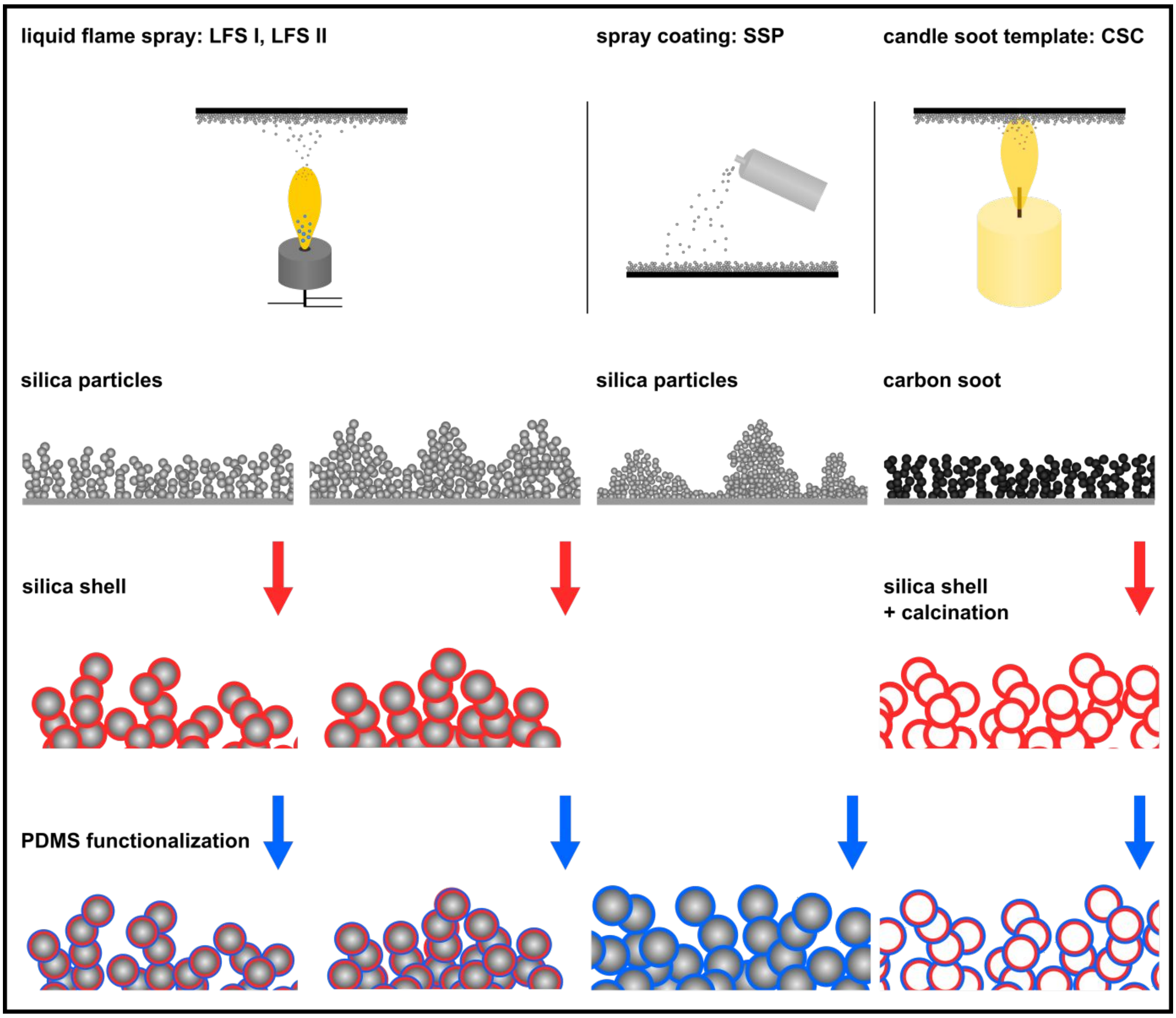

Figure S4. Schematic illustration of sample preparation: Silica particles prepared via liquid flame spray and carbon soot from a candle flame are coated with a silica shell via CVD (red). Thereafter, the candle soot template is calcinated causing combustion of the carbon core. In a final step, all samples are functionalized with PDMS (blue), rendering them super-liquid-repellent.

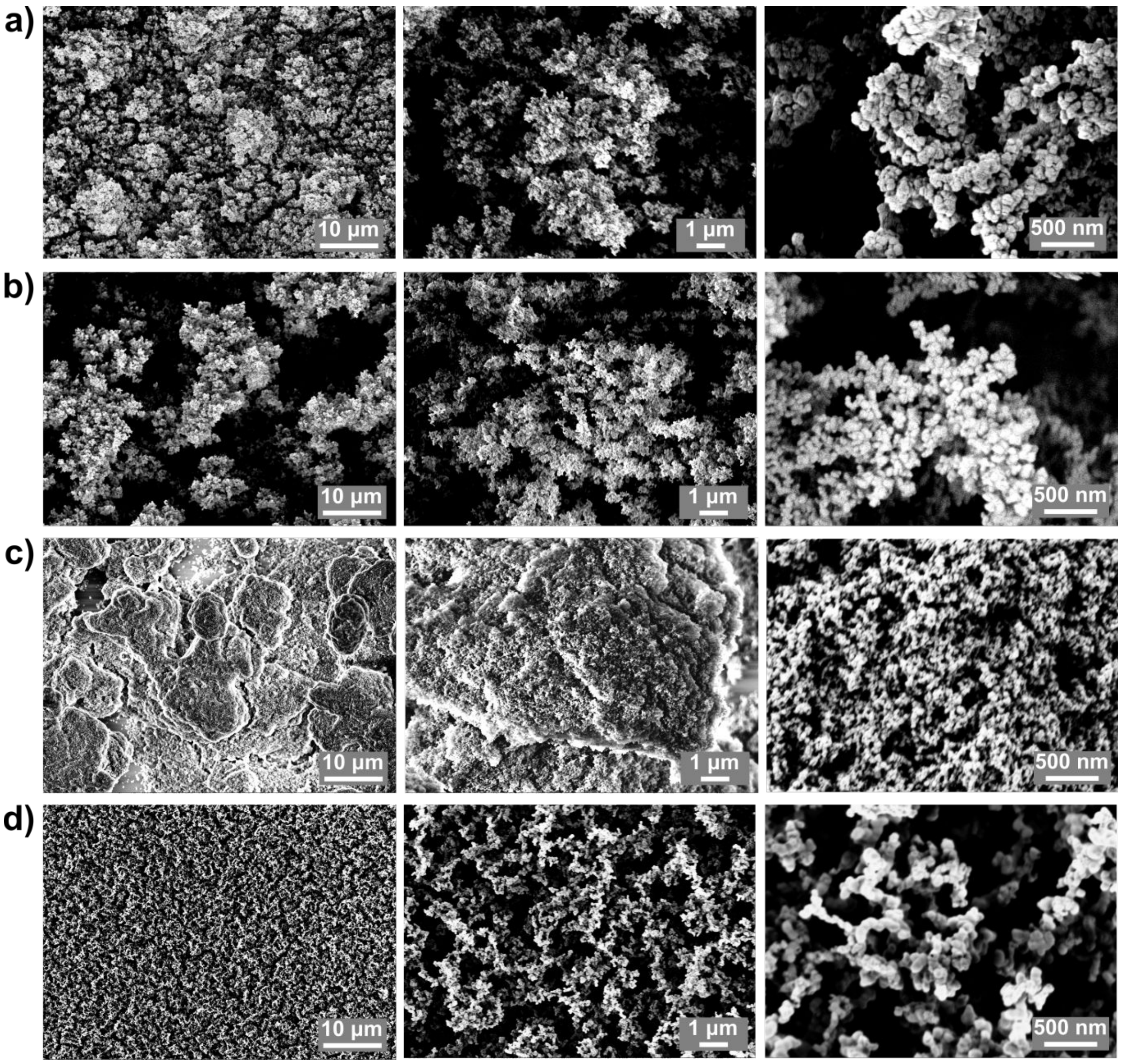

Figure S5. Top-view scanning electron microscopy images of the model surfaces at different magnifications: a) LFS I, coated for 3 min at 15 cm distance, b) LFS II, coated for 2 min at 10 cm distance, c) sprayed silica particles, and d) candle soot coating. LFS: liquid flame spray; SSP: sprayed silica particles; CSC: candle soot coating.

Prior to surface functionalization, the samples were activated with oxygen plasma (Diener Electronic Femto, 6 cm^3 min^{-1} oxygen flow rate) for 10 min at 300 W. For the PDMS

functionalization, the surfaces were placed in a desiccator (2.150 cm^3) together with 50 µL of 1,3-dichlorotetramethyldisiloxane (96 %, Alfa Aesar). The reaction was allowed to proceed at ambient temperature (21 °C) and ambient humidity (40 – 60 %) for 1.5 h. Thereafter, the samples were placed in a vacuum oven (200 mbar) for 2 h at 60 °C, to remove non-covalently bonded oligomers. For fluorine functionalization, the plasma activated surfaces were placed into a desiccator (9.200 cm^3) together with 100 µL of 1*H*,1*H*,2*H*,2*H*-perfluorooctyltrichlorosilane (PFOTS, 97 %, Alfa Aesar). The pressure was reduced to 50 mbar and the pump was turned off. After 2 h, the surfaces were removed and placed into a vacuum oven (200 mbar) for 2 h at 60 °C.

Characterization:

The morphology of the particle-based surface structures was analyzed via scanning electron microscopy. Images were acquired at a voltage of 0.5 to 3 kV using an InLens Detector, LEO 1530 Gemini, Zeiss. To reduce charging effects, the surfaces were coated with a 9 nm thick Pt layer (BalTec MED 020 Modular High Vacuum Coating System, Argon at 2×10^{-2} mbar and 30 mA).

The wetting properties of the coatings were analyzed using a contact angle goniometer (DSA100S, KRÜSS). The wettability towards different probe liquids was investigated, namely

for water, water-ethanol solutions, diiodomethane, ethylene glycol, and hexadecane. For water-ethanol solutions, the surface tension was gradually decreased by increasing the fraction of ethanol in steps of 5 wt%. Surface tensions were measured using the pendant drop method and showed good agreement with literature values[2]. The wetting properties were analyzed via the apparent static contact angle and the roll-off angle. The advancing contact angle should be close to 180°, thus too high to be measured using a contact angle goniometer.[3] The measurement of the receding contact angle on rough, super-liquid-repellent surfaces suffers from variations of the contact angle by a few degrees caused by pinning sites. Therefore, the apparent contact angle which takes a value between the advancing and receding contact angle, is determined.[4] The apparent contact angles were measured from carefully deposited 6 µL droplets. Roll-off angles were measured by placing a 6 µL droplet onto the surface and subsequently tilting the surface at a rate of 0.5 degree per second until the droplet rolled completely out of the field of view. A minimum of 6 spots was analyzed for each surface.

Drop impact was measured using water and a water-ethanol solution (35 wt% EtOH, $\gamma = 31.0$ mN m^{-1}) on a liquid flame spray I surface functionalized with PDMS and PFOTS. The substrate was tilted by 15°. Drops were produced at a height of 3.5 mm above the substrate using a gauge 32 needle. This resulted in drop volumes of approximately 4 µL and 2.3 µL for

water and the water-ethanol solution, respectively. Drop impact was recorded using a high speed camera (Fastcam AX10, Photron) and a high magnification objective (2x, Mitutoyo) at a frame rate of 5000 frames per second.

Frictions measurements:

Furthermore, we measured the friction force F_F between a drop and a surface using a dynamic adhesion force instrument (DAFI). The drop is pushed over the surface at constant velocity using a glass capillary. The friction causes a deflection of the capillary. According to Hooke's law, the force required to move the drop is equal to the deflection of the cantilever Δx multiplied by the spring constant of the capillary k_s:

$$F_F = k_s \cdot \Delta x. \quad (1)$$

If the droplet friction is dominated by contact line friction, it has been shown that the force can be calculated using Furmidge's equation. The Furmidge equation is used to calculate the force of a drop just before motion.

In analytical expressions, the three-phase contact line is often approximated by an ellipse (Figure S6).

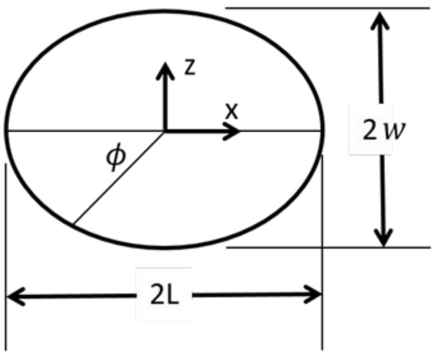

Figure S6. Sketch of the drop shape to calculate the static friction force (lateral adhesion force).

The so termed lateral adhesion force (or static friction force) follows from the integration of the horizontal component of the liquid-air surface tension γ along the three-phase contact line[5-8].

$$f = \gamma \cos\theta \cos\phi \tag{2}$$

$$F_F = -2\gamma \int_0^{\pi} r(\phi) \cos\theta \cos\phi \, d\phi$$

$$F_F = k\, w\, \gamma \left(\cos \theta_R - \cos \theta_A\right),$$

where w is the drop contact width, γ is the surface tension of the drop, and the dimensionless factor k accounts for angular variations of the contact angle around the contact line, where $k \leq 1$. θ is the contact angle, ϕ is the azimuthal angle, and r(ϕ) is the radius of the ellipse.[5] Notably, the components of the capillary force perpendicular to the direction of motion cancel out. Therefore, the width of the drop w appears in the equation of the static friction force (also termed lateral adhesion force) and not the length.

This is in line with a recent study where we verified that the width and not the length of a drop determines the static friction force. In the study, we elongated a drop parallel or perpendicular to the direction of motion. In both cases, the length of the three-phase contact line and the contact area was kept constant within experimental accuracy. For comparison, we also measured the static friction force of a sessile drop of identical volume (isotropic). We observed that the maximum static friction force greatly differs while the dynamic friction force does not depend on the initial shape of the drop (Figure S7). We observed this pronounced dependence of the static friction force on the shape of the drop for all investigated surfaces, i.e. for smooth as well as for rough surfaces.[9]

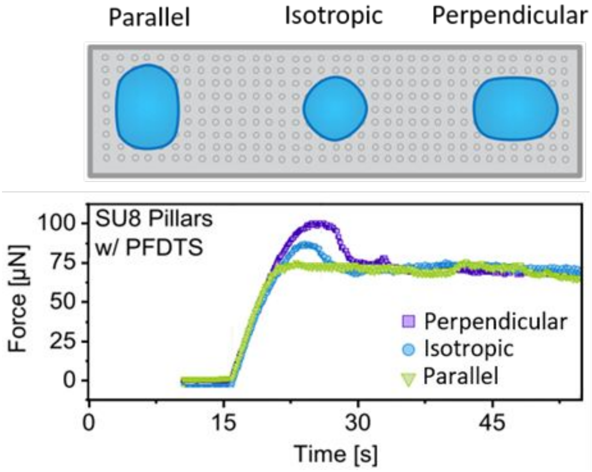

Figure S7. Top: Sketch of initial drop shapes. Force measurements on fluorinated SU-8 pillars for a constant drop volume of 3 µL. The cylindrical pillars have a diameter of 30 µm, a pillar-pillar spacing of 60 µm and a height of 5 µm. Friction force plotted with time for the different initial drop shapes: perpendicular (■), isotropic (●), and parallel (▼). Reprinted (Adapted or Reprinted in part) with permission from Laroche et al.[9] Copyright 2023 Droplet published by Jilin University and John Wiley & Sons Australia, Ltd.

When the drop is in continuous motion, friction opposes the motion. Motion also changes the contact width and the advancing and receding contact angle which become a function of velocity:

$$F_F = k \cdot w(v) \cdot \gamma \cdot \left(\cos\theta_R(v) - \cos\theta_A(v)\right). \quad (3)$$

According to equation 3, friction decreases with decreasing interfacial tension. It increases with increasing contact width. For drops on a hydrophobic surface, friction also increases for decreasing rear contact angles.

Friction forces were analyzed using water and a water-ethanol solution (20 wt% EtOH, $\gamma = 37.7$ mN m^{-1}). 15 µL drops of the respective probe liquid were placed onto the surface and brought into contact with the sensor. Then, the surface was moved at a constant velocity of 0.5 mm s^{-1} over a distance of 25 mm. The deflection of the sensor was analyzed using a Matlab script from side-view videos and the friction force was calculated from equation 1. Since droplet friction is volume-dependent, the forces were normalized to the radius of the 15 µL droplet (r = 1.5 mm from $(3V/4\pi)^{1/3}$). The radius was chosen for normalization because the contact width perpendicular to the direction of surface motion is not accessible with a goniometer. Furthermore, this is in analogy to the normalization in colloidal probe atomic force microscopy. As a sensor a glass capillary (50 mm x 0.5 mm x 0.05 mm, CM scientific Ltd.)

with a stiffness of 105 μN mm^{-1} was used. The sensor was calibrated by monitoring the deflection of the end of the glass capillary when a known load is applied. For each surface, three spots were analyzed using fresh probe liquid for each scan.

The measured friction forces (F_{exp}) were compared to values calculated according to equation 3 with $k = 1$ (F_F, Table 1). Since the contact width w is not accessible with goniometry, the contact length along the direction of surface motion in the kinetic state was used.

For both LFS surfaces and for the candle soot surface, the calculated forces slightly exceed the experimental values. There are several possible sources of error in the calculation of the friction forces: for super-liquid-repellent surfaces, the contact width and the contact length may vary by up to 20 %. Furthermore, the macroscopic advancing and receding contact angles measured from the video files may be off by a few degrees. The contact angles could be influenced by gravity and by the capillary, which may slightly pull up the droplet on the advancing side. Only in case of the spray coated surface, the calculated values are lower than the experimental values. This may be due to the pronounced stick-slip motion that does not become apparent in the advancing and receding contact angle used for the calculation.

For the LFS and the spray coated surfaces, a decrease in the friction forces in line with a decrease of the probe liquid's surface tension is observed. In case of the candle soot surface,

both the experimental and the calculated friction forces increase with decreasing surface tension due to a change in the solid-liquid interfacial area and the receding contact angle.

Table 1. Friction forces from experiments (F_{exp}) and forces calculated using equation 3 (F_F). Both values were normalized to the drop radius of $r = 1.5$ mm. Experimentally, the friction forces were determined from the deflection of a flexible glass capillary in contact with the drop while the substrate was moved at a constant velocity. The force was calculated according to equation 1. 15 µL drops of water ($\gamma = 72.0$ mN m^{-1}) and a water-ethanol solution (20 wt% EtOH, $\gamma = 37.7$ mN m^{-1}) were used as probe liquids. LFS: liquid flame spray; SSP: sprayed silica particles; CSC: candle soot coating.

Sample	γ / mN m^{-1}	F_{exp} / µN	$F_{exp}\, r^{-1}$ / µN mm^{-1}	F_F / µN	$F_F\, r^{-1}$ / µN mm^{-1}
**LFS I - PDMS**	72.0	2.7	1.8	4.6	3.0
	37.7	1.7	1.1	2.8	1.8
**LFS II - PDMS**	72.0	3.2	2.1	5.3	3.6
	37.7	1.6	1.1	1.7	1.1
**SSP - PDMS**	72.0	5.2	3.4	4.5	3.0
	37.7	3.9	2.6	2.0	1.3
**CSC - PDMS**	72.0	3.4	2.3	5.0	3.3
	37.7	5.6	3.7	5.8	3.9

Table 2. Contact angle (θ) and roll-off angle ($\theta_{roll-off}$) measurements conducted on different PDMS-functionalized surfaces. 6 μL droplets of water, water-ethanol solutions, diiodomethane (DI) and ethylene glycol (EG) were used as probe liquids. The surface tension of water-ethanol solutions was gradually reduced by increasing the ethanol fraction in steps of 5 wt% to a maximum of 35 wt%. Apparent contact and roll-off angles were recorded until the Cassie-to-Wenzel transition occurred. LFS: liquid flame spray; SSP: sprayed silica particles; CSC: candle soot coating.

	**LFS I - PDMS**		**LFS II - PDMS**		**SSP - PDMS**		**CSC - PDMS**	
γ / mN m^{-1}	θ	$\theta_{roll-off}$	θ	$\theta_{roll-off}$	θ	$\theta_{roll-off}$	θ	$\theta_{roll-off}$
72.0	170±1°	1±1°	169±1°	1±1°	169±1°	3±1°	168±2°	3±1°
57.6	170±1°	1±1°	170±1°	1±1°	169±1°	2±1°	167±1°	2±1°
46.4	169±1°	1±1°	170±1°	1±1°	168±1°	2±1°	166±1°	2±1°
41.6	169±1°	1±1°	169±1°	1±1°	169±1°	3±1°	164±1°	4±1°
37.7	169±1°	2±1°	169±1°	2±1°	168±1°	4±1°	163±2°	5±1°
34.8	169±1°	2±1°	168±1°	3±1°	166±1°	8±2°	162±2°	12±2°
32.8	167±1°	4±1°	166±1°	4±2°	< 150°		153±1°	> 10°
31.0	163±1°	7±1°	< 150°		< 150°		< 150°	
50.9 (DI)	158±1°	4±1°	160±1°	3±1°	165±1°	4±1°	164±1°	6±1°
47.7 (EG)	168±1°	2±1°	165±1°	3±1°	< 150°		165±1°	8±1°

Table 3. Contact angle (θ) and roll-off angle ($\theta_{roll\text{-}off}$) measurements conducted on different PFOTS-functionalized surfaces. 6 μL droplets of water, a 35 wt% water-ethanol solution (EtOH), diiodomethane (DI), ethylene glycol (EG), and hexadecane (HD) were used as probe liquids. LFS: liquid flame spray; SSP: sprayed silica particles; CSC: candle soot coating.

	**LFS I - PFOTS**		**LFS II - PFOTS**		**SSP - PFOTS**		**CSC - PFOTS**	
γ / mN m^{-1}	θ	$\theta_{roll\text{-}off}$	θ	$\theta_{roll\text{-}off}$	θ	$\theta_{roll\text{-}off}$	θ	$\theta_{roll\text{-}off}$
72.0 (H_2O)	166±2°	2±1°	165±1°	2±1°	169±1°	1±1°	167±1°	1±1°
50.9 (DI)	168±1°	2±1°	167±1°	1±1°	168±1°	2±1°	169±1°	1±1°
47.7 (EG)	169±1°	2±1°	167±1°	2±1°	167±1°	2±1°	168±1°	5±1°
31.0 (EtOH)	167±2°	4±1°	167±1°	7±1°	167±1°	11±1°	159±1°	23±1°
27.6 (HD)	167±1°	3±1°	163±1°	4±2°	167±1°	5±1°	163±2°	9±2°

References

(1) Deng, X.; Mammen, L.; Butt, H. J.; Vollmer, D. Candle Soot as a Template for a Transparent Robust Superamphiphobic Coating. *Science* **2012**, *335* (6064), 67–70. https://doi.org/10.1126/science.1207115.

(2) Vazquez, G.; Alvarez, E.; Navaza, J. M. Surface Tension of Alcohol + Water from 20 to 50 °C. *J. Chem. Eng. Data* **1995**, *40* (3), 611–614. https://doi.org/10.1021/je00019a016.

(3) Schellenberger, F.; Encinas, N.; Vollmer, D.; Butt, H. J. How Water Advances on Superhydrophobic Surfaces. *Phys. Rev. Lett.* **2016**, *116* (9), 2–7. https://doi.org/10.1103/PhysRevLett.116.096101.

(4) Butt, H.-J.; Liu, J.; Koynov, K.; Straub, B.; Hinduja, C.; Roismann, I.; Berger, R.; Li, X.; Vollmer, D.; Steffen, W.; Kappl, M. Contact Angle Hysteresis. *Curr. Opin. Colloid Interface Sci.* **2022**, 101574. https://doi.org/10.1016/j.cocis.2022.101574.

(5) ElSherbini, A. I.; Jacobi, A. M. Retention Forces and Contact Angles for Critical Liquid Drops on Non-Horizontal Surfaces. *J. Colloid Interface Sci.* **2006**, *299* (2), 841–849. https://doi.org/10.1016/j.jcis.2006.02.018.

(6) Furmidge, C. G. L. Studies at Phase Interfaces. I. The Sliding of Liquid Drops on Solid Surfaces and a Theory for Spray Retention. *J. Colloid Sci.* **1962**, *17* (4), 309–324. https://doi.org/10.1016/0095-8522(62)90011-9

(7) Pierce, E.; Carmona, F. J.; Amirfazli, A. Understanding of Sliding and Contact Angle Results in Tilted Plate Experiments. *Colloids Surf. A: Physicochem. Eng. Asp.* **2008**, *323* (1–3), 73–82. https://doi.org/10.1016/j.colsurfa.2007.09.032.

(8) Gao, N.; Geyer, F.; Pilat, D. W.; Wooh, S.; Vollmer, D.; Butt, H. J.; Berger, R. How Drops Start Sliding over Solid Surfaces. *Nat. Phys.* **2018**, *14* (2), 191–196. https://doi.org/10.1038/nphys4305.

(9) Laroche, A.; Naga, A.; Hinduja, C.; Aghili Sharifi, A.; Saal, A.; Kim, H.; Gao, N.; Wooh, S.; Butt, H.-J.; Berger, R.; Vollmer, D. Tuning Static Drop Friction. *Droplets* **2023**, *e42.* https://doi.org/10.1002/dro2.42
